# Nutrition-focused group intervention with a strength-based counseling approach for people with clinical depression: a study protocol for the Food for Mind randomized controlled trial

**DOI:** 10.1186/s13063-021-05279-5

**Published:** 2021-05-17

**Authors:** Johanna Roponen, Anu Ruusunen, Pilvikki Absetz, Timo Partonen, Virpi Kuvaja-Köllner, Mika Hujo, Outi Nuutinen

**Affiliations:** 1 Institute of Public Health and Clinical Nutrition, University of Eastern Finland, Kuopio Campus, Kuopio, Finland; 2 Department of Psychiatry, Kuopio University Hospital, Kuopio, Finland; 3 Deakin University, Institute for Mental and Physical Health and Clinical Translation (IMPACT), Food & Mood Centre, School of Medicine, Geelong, Victoria Australia; 4 Collaborative Care Systems Finland, Helsinki, Finland; 5 Finnish Institute for Health and Welfare (THL), Department of Public Health and Welfare, Helsinki, Finland; 6 Faculty of Social Sciences and Business Studies, Department of Health and Social Management, University of Eastern Finland, Kuopio Campus, Kuopio, Finland; 7 Faculty of Science and Forestry, School of Computing, University of Eastern Finland, Kuopio Campus, Kuopio, Finland

**Keywords:** Nutrition, Diet quality, Intervention, Trial, Solution-focused counseling, Strength-based counseling, Depression, Major depressive disorder, Randomized controlled trial

## Abstract

**Background:**

Depression is a highly prevalent mental disorder with major public health effects globally. It impairs the quality of life and reduces the ability to work and function, leading to increasing costs of sick leaves and disability pensions. Current treatment strategies focus on biological and psychological pathways while understating the role of lifestyle factors. Epidemiological studies have shown convincing evidence of an inverse relationship between diet quality and depression. However, only limited data are available on the therapeutic effects of diet quality improvement on depression. Using a randomized controlled trial design, our primary aim is to investigate the effectiveness and cost-effectiveness of a behavioral nutrition group intervention compared to a social support intervention in the treatment of depression.

**Methods:**

Participants (*N*=144, aged 20–65 years) with a diagnosis of moderate or severe depression recruited in collaboration with outpatient care units will be randomized into two arms: Food for Mind (FM) nutrition intervention (*n*=72) or Bring Good Mood (BGM) social support control group (*n*=72). Both arms will be provided with 6 group sessions over an 8-week period. FM involves improving diet quality by applying strength-based behavioral nutrition counseling and activities facilitated by a registered dietitian. The control arm comprises a befriending protocol. During the interventions, all participants will continue their treatment for depression as usual. Longitudinal data are collected at baseline, at 8 weeks, and at 6- and 12-month follow-ups. Depressive symptoms, diet quality, eating behavior, ability to work and function, and quality of life are assessed by self-reported questionnaires. A treatment expectancy questionnaire will be administered at baseline and an acceptability questionnaire at 8 weeks. The Center for Epidemiologic Studies Depression Scale is used as the primary endpoint at 1 year. The results will be analyzed with linear mixed-effects models. Economic evaluation includes both cost-effectiveness and cost-utility analysis. Two incremental cost-effectiveness ratios will be calculated to evaluate the incremental cost per QALY and the incremental cost per improvement in CES-D.

**Discussion:**

If the intervention proves to be cost-effective and acceptable, it be can be implemented in healthcare to support the treatment of depression.

**Trial registration:**

ClinicalTrials.gov NCT03904771. Retrospectively registered on 5 April 2019

**Supplementary Information:**

The online version contains supplementary material available at 10.1186/s13063-021-05279-5.

## Administrative information

Note: the numbers in curly brackets in this protocol refer to SPIRIT checklist item numbers. The order of the items has been modified to group similar items (see http://www.equator-network.org/reporting-guidelines/spirit-2013-statement-defining-standard-protocol-items-for-clinical-trials/).

**Table:** 

Title {1}	Nutrition-focused group intervention with a strength-based counseling approach for people with clinical depression: a study protocol for the Food for Mind randomized controlled trial
Trial registration {2a and 2b}.	NCT03904771, 5th April 2019 (retrospectively registered, https://clinicaltrials.gov/ct2/show/study/NCT03904771)
Protocol version {3}	08/04/2021, protocol version 2
Funding {4}	Social Insurance Institution of Finland; a project grant from the Finnish Cultural Foundation; a grant for obtaining research material
Author details {5a}	Johanna Roponen^1^, Anu Ruusunen^1,2,3^, Pilvikki Absetz^4^, Timo Partonen^5^, Virpi Kuvaja-Köllner^6^, Mika Hujo^7^, Outi Nuutinen^1^ ^1^ University of Eastern Finland, Kuopio Campus, Institute of Public Health and Clinical Nutrition, Kuopio, Finland. ^2^ Department of Psychiatry, Kuopio University Hospital, Kuopio, Finland ^3^ Deakin University, Food & Mood Centre, IMPACT Institute, School of Medicine, Barwon Health, Geelong, Australia ^4^ Collaborative Care Systems Finland, Helsinki, Finland. ^5^ Finnish Institute for Health and Welfare (THL), Department of Public Health Solutions, Helsinki, Finland. ^6^ University of Eastern Finland, Kuopio Campus, Faculty of Social Sciences and Business Studies, Department of Health and Social Management, Kuopio, Finland. ^7^ University of Eastern Finland, Kuopio Campus, Faculty of Science and Forestry, School of Computing, Kuopio, Finland
Name and contact information for the trial sponsor {5b}	No sponsors
Role of sponsor {5c}	No sponsors

## Introduction

### Background and rationale {6a}

Depression is a globally prevalent illness affecting approximately 250 million people around the world [1]. Depression causes a significant decrease in a person’s work ability and functional capacity [2–4] and also impairs the person’s quality of life [5–7]. Depression is associated with obesity [8]. In addition, the adverse effects caused by some antidepressant drugs, such as mirtazapine, are associated with a high risk of inducing weight gain [9, 10]. The economic burden caused by depression is substantial [11]. Depression is already a leading cause of disability in Europe [12] and is expected to become the leading cause of disability worldwide by 2030 [13].

According to evidence-based clinical guidelines [14, 15], depression is mainly treated with pharmacotherapy and psychotherapy. The treatment as usual seems to be effective in only one out of three cases [16, 17]. Moreover, depression is often recurrent, with relapses in at least 50% of the cases [18, 19]. It is noteworthy that the potential adverse effects associated with some antidepressants, such as weight gain, can reduce adherence to medication. In addition to the treatment as usual, the International Society for Nutritional Psychiatry Research advocates for the recognition of nutrition as an essential part of the rehabilitation of depression, as nutrition also supports the somatic well-being of patients with depression [20]. A decision on treatment should not be based solely on clinical efficacy, but on a combination of clinical efficacy and cost-effectiveness. Although there is little evidence of the financial benefits brought by a dietary intervention for treating depression, there are two recently published studies on the matter. One of them indicated that dietary treatment was highly cost-effective in treating major depression compared to a social support group program [21], while the other produced the same amount of QALYs with lower costs [22].

Despite the evidence-based association between nutrition and depression in epidemiological studies [23], the role of nutrition in the treatment of depression has thus far been primarily limited to weight management.

The benefits of the Mediterranean diet to reducing the risk of depression were already investigated in 2013 [24]. Recent reviews and meta-analyses have shown a significant association between overall diet quality and a lower risk of depression and less severe symptoms of depression [23, 25]. Such diets are high in vegetables, fruit, whole grains, seeds, nuts, and fish, while containing a limited amount of processed foods. A significant inverse association between adherence to the Mediterranean diet and the probability of depression was found in an analysis of cross-sectional studies [26], and according to the most recent meta-analysis, an improvement in diet quality can significantly reduce the symptoms of depression [27].

So far, only two randomized controlled trials have been conducted to evaluate the impact of nutrition counseling in patients who have been diagnosed with depression. The SMILES trial was the first clinical study to show that individualized nutritional counseling (7 × 1h) significantly improved diet quality, alleviated the degree of depression, increased the remission rate compared to the control group, and was feasible [28]. In the HELFIMED study, a 3-month group-based Mediterranean-style diet intervention, healthy dietary changes supplemented with fish oil were found to be beneficial for treating depressive symptoms [29]. In the same study, the intervention group participating in cooking workshops was cost-effective compared to a social support group [21].

The few previous studies have placed their focus on the impact of a dietary change on depressive symptoms. Less emphasis has been put on the mechanisms underlying a behavioral change, although other areas of research have increasingly recognized, the importance of lifestyle changes, the role of the behavior change theory, and related techniques [30].

In the Food for Mind study presented here, behavioral nutrition counseling utilizes a strength-based counseling approach, which is based on positive psychology [31] and self-determination theory (SDT) [32], and applies counseling techniques from motivational interviewing (MI) [33] and solution-focused therapy (SFT). These theories and therapeutical methods share a person-centered approach to supporting individual agency. They are described in more detail in the methods section.

## Objectives {7}

The aim of the Food for Mind study is to examine the effects of a strength-based nutrition group intervention on the symptoms of depression, diet quality, eating habits, quality of life, and work ability in patients diagnosed with depression and to evaluate its cost-effectiveness when implemented in a health care system.

The following is the primary objective:

1. To explore the effectiveness of the strength-based Food for Mind group intervention for alleviating the symptoms of depression

The following are the secondary objectives:

1. To explore whether the strength-based Food for Mind group intervention improves diet quality, eating competence, quality of life, and work ability
2. To evaluate the cost-effectiveness of the strength-based Food for Mind group intervention compared to a control group

## Trial design {8}

**Fig. 1:**
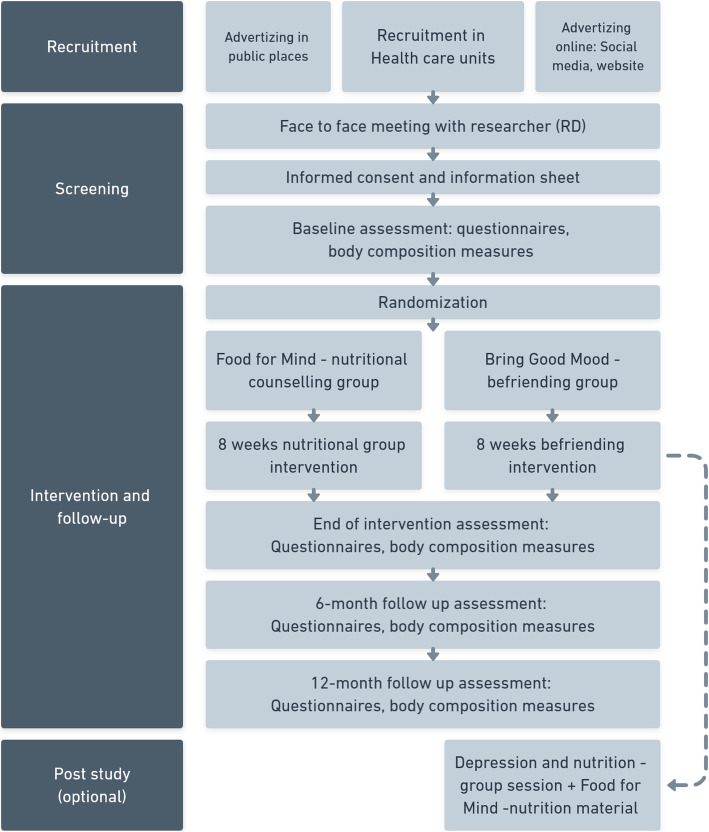
Schematic diagram of the study procedures

This study is a two-parallel group, randomized controlled clinical trial exploring the effects of a strength-based nutrition group intervention in patients diagnosed with depression. In this trial with a subject allocation ratio of 1:1, the Food for Mind intervention is expected to be superior to a social support group as a standalone intervention. The protocol is outlined in Fig. 1, and details of the assessments are given in Table 2.

## Methods: participants, interventions and outcomes

### Study setting {9}

The study will be carried out at the Institute of Public Health and Clinical Nutrition at the University of Eastern Finland in Kuopio, Finland.

### Eligibility criteria {10}

#### Inclusion criteria

Male and female participants will be eligible if they (1) are aged between 20 and 65 years, (2) have the ICD-10 diagnosis of depression (F32.1, F32.2, F32.8, F32.9, F33.1, F33.2, F33.8, F33.9, or F34.1), (3) have an individually tailored treatment and rehabilitation plan in an outpatient treatment unit, (4) are receiving treatment (medication and/or psychotherapy) that has remained unchanged for at least 2 weeks prior to randomization, and (5) are willing to participate in a group-based intervention for 8 weeks including 6 group sessions.

#### Exclusion criteria

Participants will be excluded if they (1) have a medical illness that is clinically unstable and which could be aggravated by the intervention, (2) are pregnant, (3) are participating in another nutrition or exercise intervention, and (4) have a current depressive episode or recurrent depressive disorder that is severe with psychotic features, a personality disorder, a severe eating disorder, and/or a substance abuse disorder.

### Who will take informed consent? {26a}

All participants will be recruited by a research dietitian after the informed consent process. The research dietitian will send the study description statement to eligible participants by post or email to provide potential participants with information before meeting the research dietitian. In the meeting, the research dietitian will give an informed consent form to the participants and explain this to them. The informed consent form is signed by the participants and the research dietitian. The participants have a right to withdraw from the study at any time during the research process.

### Additional consent provisions for collection and use of participant data and biological specimens {26b}

Not applicable; specimens were not collected.

### Interventions

#### Explanation for the choice of comparators {6b}

Social support is known to alleviate depressive symptoms [34]. As the Food for Mind intervention also includes a social dimension, the group will be standardized with a social support control group.

#### Intervention description {11a}

Both the FM intervention groups and the BGM control (befriending) groups meet 6 times (5 1.5-h sessions and 1 3-h session) over an 8-week period. Each group has 6 to 8 participants. For social peer support, both groups will have their own WhatsApp groups, in which participants can share their own experiences and findings. The WhatsApp mobile application is used during the intervention and for the subsequent 10 months. Both groups continue their usual treatment for depression (treatment as usual) during the study.

A written manuscript prepared for every FM group meeting drawn up together in collaboration with a behavioral scientist helps the research dietitian to stick to a cohesive plan when instructing the group. The implementation of the BGM group meetings is also harmonized by the same instructor.

#### Intervention group

The primary focus of the behavioral nutrition counseling is on improving diet quality based on the “Food for Health” Finnish Nutrition and Food Recommendations [35]. According to these recommendations, a health-promoting diet focuses on the consumption of vegetables, fruit and berries, whole-grain products, legumes, fish, vegetable oils and vegetable oil-based spreads, nuts and seeds, and fat-free or low-fat dairy products. Its composition is illustrated by the food pyramid design and single meals by the plate model. A high-quality diet has a low energy density, encompasses a need for all nutrients, and contains plenty of bioactive compounds [35]. In addition to nutritional quality, emphasis is placed on a regular meal rhythm set by breakfast, lunch, and dinner and one or two snacks as needed [35].

Behavioral nutrition counseling utilizes a strength-based counseling approach, which is based on equality and a patient-centered approach [36]. Positive psychology has identified several mechanisms that can boost health behavior, including the identification of the individual’s existing strengths increasing self-efficacy and positive affect. Learning positive thinking and applying a solution-focused rather than problem-focused approach helps finding more solutions, has direct effects on immediate mental well-being, and strengthens psychological vantage resources. Positive affect during health behavior enables enjoyment and appreciation of the process instead of just its outcome. It builds the individual’s resources and strengthens unconscious motives [31].

According to SDT, a theory of motivation and personality, people are motivated to grow and change when their universal basic psychological needs for autonomy, competence, and relatedness are fulfilled. SDT sees motivation as a continuum from the intrinsic to the extrinsic, intrinsic motivation leading to more permanent achievements. Intrinsic motivation is enhanced by reinforcing innate needs for the senses of autonomy, competence, and relatedness [37]. MI provides some useful techniques that are in line with the SDT. The most important element of MI is its collaborative, appreciative spirit that aims to understand the patient and establish rapport with her/him [38].

SFT focuses on the present circumstances, desired future, possibilities, goals, progress, and strengths [39]. The person’s sense of autonomy is enhanced by placing the responsibility for a change in the hands of individuals by giving them an opportunity to choose and set their own goals and the means used to achieve these goals. Paying attention to the individual’s skills and potential increases his/her sense of competence, and this combined with respectful and patient-centered empowering language enhances feelings of relatedness.

By utilizing basic counseling skills, including presence and listening, genuineness, empathy, positive regard, and reflection, a counselor promotes the client’s motivation, readiness, willingness, and ability to take more responsibility for his/her personal well-being and improving dietary habits.

The topic of each of the six group meetings, including activities, assignments, and actions aiming to enhance the participants’ sense of autonomy, competence, and relatedness, is described in Table 1. In summary, each meeting includes discussions on the topic of the session; action-based methods, such as grocery shopping, cooking, eating together, and using all senses; and completing home assignments aiming to elucidate how the participants can improve their diet quality in their daily lives in practice. Each new meeting starts with the participants providing feedback on their experiences and positive changes, if any, in their eating habits resulting from completing the home assignments. In each group meeting, participants receive printed counseling material and assignments related to the day’s topic. The material and assignments aim to support the group participants to observe their eating behavior and practice mindful eating to identify hunger and satiety and pinpoint how they are feeling. The intervention group sessions are facilitated by the research dietitian. The protocol of each nutrition counseling meeting is presented in Additional file 1 (see Additional file 1).

**Table 1:** Activities, assignments, and actions to enhance participants’ sense of autonomy, competence, and relatedness

Meeting topics and learning objectives (LO)	Activities	Home (HA) and WhatsApp (WA) assignments	Sample actions to enhance the sense of autonomy, competence, and relatedness
1. Getting to know each other: - Grouping - Finding strengths LO: “We’ll get to know each other, and I feel like I’m an important part of the group.”	1. Finding personal strengths 2. Sharing thoughts of personal motives to participate in the FM group Group discussion: nutrition and depression—how eating affects your mood	HA: keeping a record of health-promoting eating habits during good and bad days WA: sharing a photograph of one personally meaningful thing	Autonomy: finding out the participants’ important/meaningful reasons/motives for participating Competence: identifying existing knowledge of the topic Relatedness: noticing and respecting individual experiences, giving and receiving social support
2. Diet quality and meal frequency: - Identifying health-promoting characteristics in diet - Setting realistic goals LO: “I’ll understand the meaning of meal rhythm and diet quality to my wellbeing.”	1. Experiences of differences in diet and eating between good days and bad days 2. Goal setting	HA: focusing on meal frequency for a week HA: evaluation of ones’ own eating patterns WA: sharing favorite starter, main dish, and dessert chosen from the Food for Mind—recipe booklet	Autonomy: encouraging/helping participants to choose and set realistic and meaningful goals of their own and to plan actions that they will be committed to in achieving them. Competence: noticing and strengthening what is already good in the participants’ current habits and helping them plan concrete actions leading them to achieve their goals Relatedness: respecting differences in the participants’ habits and experiences, making use of this diversity when promoting change, giving and receiving social support
3. Nutrition and depression—evidence-based knowledge: - Identifying existing positive characteristics in meal frequency and eating habits LO: “I’ll understand evidence-based knowledge about the association between depression and diet/nutrition.”	1. Experiences and findings about meal frequency and its effects on good mood—discussion in pairs and in group Group discussion: nutrition and depression utilizing evaluations of participants’ own eating patterns	HA: adding one food item known to affect depression positively to your diet HA: getting familiar with mindful eating WA: participants sharing a picture of a food item or a dining experience, which has made them feel good	Autonomy: introducing several options of food items with a positive effect on depression and offering the participants freedom of choice Competence: noticing and strengthening the factors already good in the participants’ current habits and helping them make sustainable choices for new habits Relatedness. utilizing team/groupwork to enhance team spirit and to give and receive social support
4. Food for Mind in practice: - Practicing mind-friendly cooking - Familiarization with mindful eating LO: “I’ll learn to prepare easy and mind-friendly food.”	1. Group exercise about mindful eating and different hunger types using a mind map 2. Cooking, utilizing mindfulness 3. Eating together applying mindful eating	HA: existing food choices in the participants’ diet WA: sharing a photograph of a situation, in which a participant has utilized or could utilize mindfulness in the future	Autonomy: creating an atmosphere that supports participants’ sense of autonomy Competence: training on new skills (new cooking skills), strengthening existing (cooking) skills Relatedness: utilizing team/groupwork to enhance team spirit and to give and receive social support
5. Food for Mind food items in a grocery store—navigation: - Getting familiar with a mind-friendly food selection LO: “I’ll get new tips to my food choices.”	1. The participants’ experiences and observations of their own food choices 2. Getting familiar with the selection of Food for Mind food items available in the grocery store	HA: focusing on meal frequency for a week WA: sharing a photograph of one mind-friendly food item each participant has adopted in diet	Autonomy: learning to read labels to make personally relevant and acceptable choices Competence: giving positive feedback about what is already good in the participants’ current food choices, learning to read and understand labels, and using this knowledge in one’s daily life Relatedness: utilizing pair/team/groupwork to enhance team spirit and to give and receive social support
6. Tools for the future: - Discovering and ensuring to the tools the participants have for putting the dietary goals into action and successfully maintaining them LO: “I’ll notice my success and I’ll find ways to enhance my mood.”	1. A gallery walk-through method for group success, benefits, support, and future prospects with group discussion 2. Group discussion: hopefulness and gratefulness	Future exercises - Gratefulness exercise - Letter for the future exercise	Autonomy: highlighting the active and autonomous role participants have played in selecting and making lifestyle choices that have improved their health and well-being Competence: positive feedback on personal and group success Relatedness: giving and receiving social support

#### Control group

The BGM control group follows a befriending protocol [34] and has a visit schedule identical in its content and length with the FM intervention group. The befriending group consists of a discussion of neutral topics of interest, such as hobbies, music, and sports, and engaging in activities together, with the intention of keeping participants engaged and in a positive mood. Befriending groups are facilitated by a rehabilitation counselor from a non-profit association (Turvalinkki ry), which organizes activities open for anyone with a focus on considering the needs and wishes of mental illness rehabilitees.

### Criteria for discontinuing or modifying allocated interventions {11b}

There will be no special criteria for discontinuing or modifying allocated interventions.

### Strategies to improve adherence to interventions {11c}

Diet quality is assessed with the Index of Diet Quality and Diet Frequency Questionnaire at the baseline, at the end of the intervention, at the 6-month follow-up, and at the end of the study (12-month follow-up) to monitor the adherence with the intervention.

### Relevant concomitant care permitted or prohibited during the trial {11d}

All participants will receive treatment as usual as part of their psychiatric treatment unaffected by this study or the group in which they participate.

### Provisions for post-trial care {30}

At the end of the study, the participants in the BGM group will receive the same Food for Mind nutrition counseling material as the participants in the FM group, and they have an opportunity to participate in one depression and nutrition group session (1.5 h) facilitated by the research dietitian.

### Outcomes {12}

#### Primary outcomes

The primary outcome (depression) is a change in the CES-D Depression Scale [40] scores from the baseline to the 12-month follow-up.

A clinically relevant change is defined as (a) an at least 50% decrease in the CES-D Depression Scale score and (b) a total score undercutting certain limit values. In addition to the typical cutoff score of 16, cutoff points of 12 [41] and 22 [42] are used in the analysis.

#### Secondary outcomes

The secondary outcomes (diet quality, eating competence, quality of life, work ability and functional capacity, cost-effectiveness) include the following:

- Changes in the Index of Diet Quality (IDQ) [43, 44] from baseline to 12 months
- Changes in the Satter Eating Competence Inventory 2.0 (ecSI 2.0™) [45] and the Three-Factor Eating Questionnaire-R18 (TFEQ-R18) [46] from baseline to 12 months
- Changes in the Assessment of Quality of Life (AQoL-8D) [47] from baseline to 12 months
- Changes in the work ability and functional capacity questionnaire [48, 49] from baseline to 12 months
- The difference in quality-adjusted life years (QALYs) between the intervention group and the control group.
- The incremental cost-effectiveness ratio (ICER) [50] as well as the incremental cost-utility ratio (ICUR) [50] of the intervention compared to the control group.

#### Follow-up outcomes

Follow-up outcomes are changes in the primary and secondary outcomes from baseline to the 8-week and 6-month follow-up.

Other pre-specified outcomes are as follows:

- Treatment expectancy questionnaire: As treatment expectations may influence the outcome, they will be evaluated with the treatment expectancy questionnaire [51] and taken into account in the statistical analysis.
- Acceptability: The acceptability of the Food for Mind group rehabilitation program will be evaluated with the acceptability questionnaire based on the Theoretical Framework of Acceptability (TFA) [52].
- Changes in weight, body mass index (BMI), body fat, and fat-free body mass as measured with a body composition analyzer (InBody 720) from baseline to 12 months.

### Participant timeline {13}

Figure 1 shows the schematic diagram of study procedures.

### Sample size {14}

The total sample size of *n*=144 is based on the power calculation, when *α*=0.05, power=0.8, and effect size=0.5, and the estimated loss of participants is 15%. The calculation is based on the decline of 7 points assessed with the Center for Epidemiologic Studies Depression (CES-D) Scale.

### Recruitment {15}

Recruitment will be conducted in collaboration with ten public and private health care service providers (public health and occupational health care) in Northern Savo, near the University of Eastern Finland’s Kuopio Campus. The principal investigator and the research dietitian will introduce the study to the health care staff (doctors, nurses, psychologists) as part of their clinical team meetings in all recruiting organizations and will distribute tailored brochures targeted separately at health care staff and patients. The staff are also given a suggestion of how to introduce the study to potentially eligible patients. These instructions were developed by the behavioral scientist and the principal investigator of the research team.

The staff will select potentially eligible patients according to the diagnostic criteria, introduce the study to the patients with the material described above, and inquire them about their willingness and readiness for participation. Based on the participants’ consent, their contact information is provided to the research dietitian for further eligibility screening.

The visibility of the study will also be increased by placing posters with general information about the study on notice boards for the purpose of collaborative recruitment at health care units, public libraries, and cafes and by publishing Facebook ads and sending emails in co-operation with local associations. Information about the study is also available online (www.ruokaamielelle.fi) and on a social media platform (Facebook).

### Assignment of interventions: allocation

#### Sequence generation {16a}

Participants will be randomized either into the FM behavioral nutrition intervention group (*n*=72) or the BGM control (befriending) group (*n*=72) in a 50:50 ratio using block randomization, which is a commonly used technique in clinical trial design [53].

Each pair of the starting intervention and control small groups (*n*=12–16) will form one block. A random study number between 1000 and 9999, and a number coding the treatment (0 = control, 1 = intervention) will be generated with Microsoft Excel for each study participant. Treatment numbers will be generated in a way that ensures that the setup becomes balanced.

#### Concealment mechanism {16b}

To prevent forehand knowledge of the allocation sequence, randomization is done separately for each block (*n*=12–16) after obtaining the participants’ consent and conducting baseline assessments. With this procedure, the aim is to ensure that prior information does not selectively influence the choice of subjects.

#### Implementation {16c}

The research dietitian will register eligible participants after obtaining consent at a face-to-face meeting. A data manager not connected to the study will perform the randomization and send the random numbers and group assignment directly to the research dietitian by email, who provides information about the allocation results to the participants by SMS and email including the meeting schedule and group program. If a person is unwilling to participate in the BGM control group after being randomized into it, she/he will be encouraged to participate by the research dietitian, highlighting that all participants in the BGM group will have an opportunity to participate in one Food for Mind nutrition meeting and receive the Food for Mind counseling material at the end of the study. Naturally, there is an option to refuse to participate in the study.

### Assignment of interventions: blinding

#### Who will be blinded {17a}

Due to the limitations in research resources, we will not be able to blind the care providers or outcome assessors. This is unlikely to introduce bias as the main outcome measure is the self-administered questionnaires, filled out by the participants. To avoid confirmation bias, participants’ data will be re-coded for statistical analysis by a data manager not related to the study, or if possible, the data will be analyzed by an external data analyst.

#### Procedure for unblinding if needed {17b}

An open-label design is used to avoid unblinding.

## Data collection and management

### Plans for assessment and collection of outcomes {18a}

The questionnaires and methods used for the assessments are presented in Table 2. Self-administered AQoL-8D and treatment expectancy questionnaires have been double-translated from English into Finnish.

**Table 2:** Assessment time points

Questionnaire/method	0	8weeks	6months	12months
Baseline characteristics				
Age, sex, education, occupation, working time pattern, marital status, household size, smoking, alcohol use, special diet	x			
Diagnosis of depression, year of first diagnosis	x	x	x	x
Medication	x	x	x	x
Employment status	x		x	x
Distance to group meeting facilities, mode of transportation	x			
Depression				
Center for Epidemiologic Studies Depression (CES-D) Scale	x	x	x	x
Seasonal Pattern Assessment Questionnaire (SPAQ)	x			
Quality of diet				
The Index of Diet Quality (IDQ)	x	x	x	x
Diet Frequency Questionnaire	x	x	x	x
Household management questionnaire	x	x	x	x
Eating behavior				
*Three-Factor Eating Questionnaire-r18* (TFEQ-r18)	x	x	x	x
Satter Eating Competence Inventory (ecSI 2.0™)	x	x	x	x
Quality of life				
Assessment of Quality of Life (AQoL-8D)	x	x	x	x
Ability to work and function				
Ability to work and function questionnaire	x	x	x	x
Cost-effectiveness				
Distance and mode of transportation to group meetings	x			
Costs of intervention				x
Costs of participants’ time use				x
Cost of participants’ travel				x
Treatment expectancy				
Treatment expectancy questionnaire	x			
Acceptability				
Theoretical Framework of Acceptability (TFA)		x		
Weight and body composition				
Body composition analyzer (InBody720)	x	x	x	x

#### Depression

The CES-D depression scale is a short self-report scale designed to measure depressive symptoms [40]. It includes 20 items, all scored from 0 (zero) to 3 points resulting in the total score ranging from 0 (zero) to 60 points. Four items (#4, #8, #12, #16) are reversed before calculating the total score. If information is missing for more than 5 items, the total score will not be calculated. Otherwise, the sum variable is calculated as follows: the sum of the answered items is divided by the number of answered items, and the number thus obtained is multiplied by 20. The higher the total score is, the more depressive symptoms the individual experiences.

The CES-D is supplemented with the Global Seasonality Score (GSS) calculated from a modified version [54] of the original Seasonal Pattern Assessment Questionnaire (SPAQ) [55, 56], which is a self-report tool used to evaluate the magnitude of seasonal changes in mood, appetite, weight, sleep duration, social activity, and energy level.

#### Diet quality

Diet quality is assessed with the validated Finnish Index of Diet Quality (IDQ) [43, 44] instrument. It evaluates the implementation of a health-promoting diet and nutritional recommendations by using 18 questions about the consumption of whole grains, fat, vegetables and fruit, sugar, and dairy products, as well as a regular meal rhythm. Based on the responses, the diet is scored from 0 (zero) to 15, and a score of 10 or higher indicates a health-promoting diet. The IDQ can also be used as a continuous variable without a cutoff into a health-promoting or unhealthy diet.

Description of diet quality is supplemented with the 11-item Diet Frequency Questionnaire (FFQ) [57], which contains questions about the meal rhythm, menu planning, cooking at home, eating out or social eating, use of dietary supplements, and food security. The frequency of eating fatty fish, nuts, seeds, seed oils, and snacks (chips, cookies, icecream, etc.) was added to the questionnaire for more detailed information on the foods suggested to be associated with depression. The FFQ intakes of these items will be converted to daily frequency equivalents (DFE) which are calculated by allocating the proportional values to the original frequency categories with reference to a base value of 1.0, equivalent to “once a day” [58, 59].

#### Eating behavior and competence

The psychology of eating behavior, including the cognitive, behavioral, emotional, and social aspects of eating habits, is involved in individuals’ adherence to changes in their dietary habits. The Three-Factor Eating Questionnaire R18 (TFEQ-R18) evaluates cognitive restraint (6 questions), uncontrolled eating (9 questions), and emotional eating (3 questions) [46]. In this study, from the point of view of the depressed individuals, emotional eating referring to a tendency to eat in response to negative emotions is more relevant in comparison with cognitive restraint and uncontrolled eating as supported by the association between depressive symptoms and emotional eating [60–63].

Responses of TFEQ-R18 are scored on a 4-point scale; the mean values are calculated for each factor, and these are subsequently transformed to correspond to the total score of 0 (zero) to 100. The results are calculated as a percentage of the highest possible value, between 0 and 100%, with higher values indicating greater engagement in the behavior. The higher the value is for each factor, the more likely it is to affect eating behavior.

In addition to the TFEQ-R18, eating competence is measured with the 16-item Satter Eating Competence Inventory (ecSI 2.0™) instrument [45] based on the Satter Eating Competence Model (ecSatter) [64, 65]. ecSatter is a biopsychological model designed for use in nutrition education and the characterization of eating attitudes and behavior. In adults [66] and adolescents [67], eating competence is associated with higher diet quality.

ecSI 2.0™ evaluates 4 dimensions related to food and eating. First, eating attitudes (5 items) comprise a positive, relaxed, and flexible interest in food and eating and a responsive attunement to the inner and outer experiences relative to eating. Second, food acceptance (3 items) means cognitive and behavioral processes and external influences of learning to accept and like a variety of foods, including new foods. Third, internal regulation of food intake (3 items) refers to the experiential processes of hunger, appetite, and satiety. Fourth, management of eating context (5 items) prioritizes the structure and meal planning as well as permission to eat adequate amounts of the preferred food at predictable times [45, 66]. All items are scored on a Likert scale and assigned values of 0 (zero) to 3. The scores of each subsection are summed up into an overall score of 1 to 48. The scoring follows the rationale of Garner [68]. The cutoff score for eating competence is 32 points.

#### Quality of life

The AQoL-8D [47] is a self-report questionnaire containing 35 questions which are grouped into 8 dimensions, including independent living, happiness, mental health, coping, relationships, self-worth, pain, and senses, which can be grouped into 2 super-dimensions: physical dimension and mental/physiological dimension. The scores on the AQol-8D both as a “psychometric” health-related quality of life (HRQoL) and a “utility” measure will be calculated with the scoring algorithms available via www.aqol.com.au. A simple psychometric score for health-related quality of life (HRQoL) is derived by adding the unweighted response order of each question with a total score of 35 to 177. Profile scores are calculated for the different dimensions. The AQoL-8D is also used as a “utility measure” for a cost-utility analysis requiring the computation of quality-adjusted life years (QALYs).

#### Work ability and functional capacity

The work ability and functional capacity questionnaire includes 3 modified questions focusing on the respondent’s work ability [48], including self-assessment of ability to work or study on a scale from 0 (zero) to 10 where 0 equals unable to work and 10 equals the best ability to work; evaluation of work ability in terms of the physical, physiological, and social requirements of the current job; and assessment of the probability of sustaining work ability until retirement age, and three 5-point scale questions about functioning in social situations, including loneliness [69], ability to do things together with others, and ability to confront strangers and address issues with them [70].

#### Economic evaluation

Two economic analyses will be carried out: a cost-utility analysis (CUA) [50] and cost-effectiveness analysis (CEA) [50]. Two ICERs will be calculated to evaluate the incremental cost per QALY (calculated from AQoL-8D scores) and the incremental cost per improvement in CES-D. An improvement is considered to occur if there is a 50% decrease in the CES-D depression scale score, which is a clinically relevant change, and also when the score falls below 12, 16, or 22 (“no depression”). The incremental cost-effectiveness ratio describes an extra cost in relation to an extra effect.

The viewpoint of the analyses does not cover the entire society, as has been recommended, because information on health care service use is not available for this analysis. The analysis will be conducted from the viewpoint of intervention providers, which includes the costs of the intervention such as the salaries of the staff with their ancillary costs, the costs of the facilities (room rental, nutrition guidance material), and the resources used by the participant for treatment (time, travel costs).

A number of sensitivity analyses will be carried out to assess the changes in variables and parameters with the greatest uncertainty or with the greatest impact on the total costs. Costs will be presented in 2020 prices in euros. Discounting is not needed due to the short run of the intervention.

#### Treatment expectancy

Treatment expectations can influence the intervention outcome. Thus, treatment expectations will be evaluated with the 6-item Treatment Expectancy Questionnaire [51]. It examines the extent to which participants believe that the treatment will help. Four questions are related to thinking, and two are related to feeling. Standardized scores will be used due to measures with 2 different rating scales (1 to 9 and 0 to 100%).

#### Acceptability

Acceptability [52] can have an impact on the treatment outcome and is therefore taken into account in this study. The acceptability of the Food for Mind group intervention will be evaluated with a self-evaluation questionnaire based on the Theoretical Framework of Acceptability (TFA) [52]. According to Sekhon et al., TFA is a multi-faceted construct that reflects the extent to which people delivering or receiving a healthcare intervention consider it to be appropriate, based on anticipated or experimental cognitive and emotional responses to the intervention. In TFA, the 7-component construct of acceptability is represented as follows: (1) affective attitude (“how an individual feels about the intervention”), (2) burden (“the perceived amount of effort that is required to participate to the intervention”), (3) ethicality (“the extent to which the intervention has a good fit with an individuals’ value system”), (4) intervention coherence (“the extent to which the participant understands intervention and how it works”), (5) opportunity costs (“the extent to which benefits, profits or values must be given up to engage in the intervention”), (6) perceived effectiveness (“the extent to which the intervention is perceived as likely to achieve its purpose”), and (7) self-efficacy (“the participants’ confidence that they can perform the behavior(s) required to participate in the intervention”). In addition to the acceptability questionnaire, personal feedback on the solution-focused facilitation of 6 group meetings will be collected by 5 questions adapted from Sharry [71]. All questions are scaled from 1 (completely disagree) to 5 (completely agree).

#### Weight and body composition

Weight (kg), BMI (kg/m^2^), body fat (%), and fat-free body mass (kg) are measured with the InBody 720 body composition analyzer (Manufacturer InBody Co., Ltd., Korea), which utilizes the bioelectrical impedance analysis (BIA) method.

### Plans to promote participant retention and complete follow-up {18b}

To promote participant retention, the research dietitian and control group counselor will remind participants before each group meeting by sending personal emails or a message to their WhatsApp groups. The research dietitian will send personal SMSs to the participants to remind them of the study follow-up appointments.

### Data management {19}

Data collected in paper format will be stored in a locked cabinet in a secure facility at the University of Eastern Finland and will only be accessible to the authorized research personnel. Data collected electronically will be stored (in a re-identifiable format) on servers securely housed and managed by the University of Eastern Finland. Any transmission of web-based data is encrypted. All data will be stored on a private, firewall-protected network. Study personnel will be given individual user IDs and passwords. Access will be restricted on a role-specific basis. After completing data cleaning measures, the database will be closed. All data will be exported to appropriate software to enable statistical analysis.

### Confidentiality {27}

All study data and research results are processed in line with the EU General Data Protection Regulation (679/2016). The confidentiality and anonymity of all personal data will be ensured throughout the study.

Each participant will be given a numeric identification code, which will be used to identify their data. The list of participants with their identification codes will be stored electronically on the university’s servers and will be password-protected. The results of this study will be analyzed and reported at the group level. Individual participants cannot be identified from the results.

Research data and documents will be stored for 6 years, after which they will be destroyed either by shredding the paper files or deleting electronic files. Data collected based on consent will not be used in subsequent studies.

### Plans for collection, laboratory evaluation, and storage of biological specimens for genetic or molecular analysis in this trial/future use {33}

Not applicable; no specimens were collected.

## Statistical methods

### Statistical methods for primary and secondary outcomes {20a}

The effects of the intervention on the primary and secondary outcomes will be analyzed following the intention-to-treat (ITT) principle. Group differences will be estimated using linear mixed effects models which allow considering the nested dependency structure generated from small groups and small group counselors in the data. All tests will be conducted using an alpha level of 0.05 and reporting 95% confidence intervals. Statistical analysis will be performed using the R software [72] and IBM SPSS Statistics® (IBM Corp., Armonk, NY, USA).

### Interim analyses {21b}

Not applicable; no interim analysis was performed.

### Methods for additional analyses (e.g., subgroup analyses) {20b}

The effects of the intervention on the primary and secondary outcomes will also be analyzed with additional per-protocol analysis with the participants who attended all 6 group meetings.

### Methods in analysis to handle protocol non-adherence and any statistical methods to handle missing data {20c}

Non-adherence will be handled with the use of the ITT principle in the analysis, by which we aim to avoid the effects of potential study dropouts and protocol deviations. The analysis will be made with the complete parts of the data for each statistical unit, and incomplete parts will be omitted. Missing data will be handled with suitable methods after evaluating the mechanism [73] under which the missing data occurs.

### Plans to give access to the full protocol, participant level-data, and statistical code {31c}

The datasets analyzed during the current study are available from the corresponding author on reasonable request.

## Oversight and monitoring

### Composition of the coordinating center and trial steering committee {5d}

The Food for Mind study is considered a low-risk trial designed by an experienced multidisciplinary research team, under whose guidance the study will be managed without the need for a steering committee. The research dietitian and principal researcher are responsible for the trial’s day-to-day activities, while the recruitment process is carried out in collaboration with the staff of health care providers. The co-operating units are regularly informed about the recruitment process and future interventions. The research group plays a supporting role. The University of Eastern Finland provides organizational support, including statistical expertise. The ethics committee is informed about any possible changes in the study protocol.

### Composition of the data monitoring committee, its role, and reporting structure {21a}

This strength-based nutrition group intervention is founded on the “Food for Health” Finnish Nutrition and Food recommendations to improve diet quality without any risks to the adult study population. Therefore, establishing a data monitoring committee (DMC) was considered unnecessary

### Adverse event reporting and harms {22}

This is a low-risk trial, and adverse events and harms are not anticipated nor monitored. However, if psychological distress is noticed in relation to the group sessions, the participants are guided to discuss it in their treatment unit.

### Frequency and plans for auditing trial conduct {23}

After the approval, the ethics committee will be informed about any changes made to the study protocol. Additionally, the publications made based on the trial will be sent to the ethics committee. The project management team has regular meetings once every 2 months and keeps in touch as necessary between regular meetings.

### Plans for communicating important protocol amendments to relevant parties (e.g., trial participants, ethical committees) {25}

Any changes made to the study protocol will be communicated to the ethical committee.

## Dissemination plans {31a}

The results of the study will be published in three original articles, which, in turn, will be part of a doctoral thesis. Once published, the abstract of the dissertation and personal feedback of the results will be sent to the study participants.

After the end of the study, the nutrition intervention model will be available in the National Innokylä Online Innovation Community and in the Health Village web service of Helsinki University Hospital (HUS) (www.mentalhub.fi) for specialized health care. Information about the availability of the model will be provided through the national educational and professional interest organization for nutritionists (the Association of Clinical and Public Health Nutritionists in Finland, RTY).

If the rehabilitation model was cost-effective and acceptable, it can be implemented in health care as part of the health care services for depression.

## Discussion

Preliminary evidence from two previous interventions supports the effects of diet quality on depressive symptoms [28, 29]. This RCT nutrition group intervention study increases evidence-based knowledge on the effectiveness of diet quality in the treatment of depression as well as on its acceptability and cost-effectiveness.

This document describes the protocol of a randomized clinical trial examining the effects of diet quality into symptoms of depression with an 8-week group-based behavioral nutrition counseling program. The theory-based counseling method generates a novelty value as the Food for Mind study is the first RCT which utilizes a strength-based counseling method to improve the diet quality of patients diagnosed with depression. It has been noticed that patient-centered counseling, which supports autonomy and strengthens motivation, self-control, and competence, is likely to result in more long-term changes compared to expert-oriented counseling [30, 32]

The Food for Mind intervention was designed to be implementable in health care as a part of the treatment of depression involving the application of brief therapy methods, such as meeting only six times during an 8-week period. As with making lifestyle changes and forming new habits in general, introducing and maintaining dietary changes may take a long time [74], depending on the habit in question and subjective capability to develop new habits. Therefore, our original plan was to use social media, namely the WhatsApp mobile application, after the intervention to encourage dietary improvements, answer the participants’ questions, and utilize social support. However, acknowledging the limited resources of registered dietitians working in health care, in practice, it may not be possible for them to facilitate an active social peer group chat for a year. Similarly, the active use of the application was deemed overly demanding by our research environment due to having two parallel WhatsApp groups: the intervention and the control group. Therefore, the role and use of WhatsApp in this context need to be re-evaluated.

The process of recruiting study participants for a randomized clinical trial has been found to be challenging in the population of individuals with depression [75], as was also found in a randomized controlled SMILES trial of dietary improvement for adults with major depression [28]. Group activities are increasingly used in the treatment of depression in health care but may be too demanding for some persons with depression. Therefore, the group sizes of this study are kept suitably small. In addition, the subjects’ thoughts about the suitability of the group intervention to them and their readiness about participating are discussed in the first meeting with the research dietitian. Further, the health care staff may be unfamiliar with nutrition care as part of the treatment of depression, and recruiting eligible patients for the study may require a particular interest in bringing up the topic of participation in the study. To facilitate the recruitment, we have produced material to be used in recruiting staff. Moreover, the randomization of participants to the intervention and control groups might discourage some patients from participating in the study, even though they will get an option to have social peer support in the control group, to take part in one nutrition meeting, and get all the printed material at the end of the study.

The course of research has been delayed for more than 6 months because of the global COVID-19 pandemic. The priority of outpatient care units has been on primary care, which has limited the time left for recruiting patients. In addition, national restrictions to group gatherings and the closure of research facilities due to the pandemic have caused an interruption to recruitment and running group sessions. The strengths of this study include the study design (RCT) and the recruitment of study participants in collaboration with outpatient care units confirming the participants’ depression diagnosis and ongoing treatments. Moreover, the study utilizes behavior change theories as applied in a strength-based counseling method to achieve improvement in diet quality, and the effect of social peer support is controlled with the “befriending” control group.

The study has two main limitations, which may undermine the generalizability of the findings. First, as double blinding is not possible, the data will be re-coded for the analyses. Second, due to financial limitations, weight and body composition are measured and data from the questionnaires are collected by the research dietitian.

In summary, there is a need for novel treatment approaches for depression to support medication and psychotherapeutic interventions to improve both the physical and mental well-being of patients.

## Trial status

Recruitment began on 1 February 2018, and it will be continued until the end of 2022 (protocol version 2).

## Supplementary Information

**Additional file 1.** The Protocol for All Food for Mind Group Sessions.

## Data Availability

Only the members of the Food for Mind study group have access to the data during the study.

## Abbreviations

AQoL-8D: Assessment of Quality of Life
BGM: Bring Good Mood
BMI: Body mass index
CES-D: Center for Epidemiologic Studies Depression
CHD: Cardiac heart disease
CUA: Cost-utility analysis
DFE: Daily frequency equivalents
ecSatter: Satter Eating Competence Model
ecSI 2.0™: Satter Eating Competence Inventory
FFQ: Diet Frequency Questionnaire
FM: Food for Mind
GSS: Global Seasonality Score
HE: Home exercise
HRQoL: Health-related quality of life
HUS: Helsinki University Hospital
ICD-10: International Statistical Classification of Diseases and Related Health Problems
ICER: Incremental cost-effectiveness ratio
ICUR: Incremental cost-utility Ratio
IDQ: Index of Diet Quality
ITT: Intention-to-treat
MI: Motivational interviewing
PUFA: Polyunsaturated fat acid
RD: Registered dietitian
RTY: The Association of Clinical and Public Health Nutritionists in Finland
SDT: Self-determination theory
QALY: Quality-adjusted life years
SFT: Solution-focused therapy
SPAQ: Seasonal Pattern Assessment Questionnaire
TFA: Theoretical Framework of Acceptability
TFEQ-R18: Three-Factor Eating Questionnaire R18
