# Supplementary material for: Nutrition-focused group intervention with a strength-based counseling approach for people with clinical depression: a study protocol for the Food for Mind randomized controlled trial
Source: Trials. 2021 May 17;22:344. doi: 10.1186/s13063-021-05279-5 (PMC8127236; doi:10.1186/s13063-021-05279-5)
Supplement: Supplementary file 1 — Additional file 1. The Protocol for All Food for Mind Group Sessions. [file 13063_2021_5279_MOESM1_ESM.docx]

Attachment. Strength-based nutrition group intervention for people with clinical depression: a study protocol for the nutrition counselling for the Food for Mind randomized controlled trial

**The Protocol for All Food for Mind Group Sessions**

Before each group meeting, I as a counsellor prepare myself for the session by reflecting on the following aspects:

- I have positive expectations for the group
- I could make everyone feel welcomed in the group
- I have patience for quiet moments and give each group member enough time to speak up. Everyone gets to have a say.
- I remember that group members’ experiences, not mine, lay at the heart of the activities.
- I show courage in using participatory methods to ensure a good level of energy in the group throughout the meeting.
- I help highlighting something positive in everyone’s activities.
- Everyone will get support and encouragement.
- Did I tell the group that I trust them?
- I strengthen everyone’s willingness to stay in the group.
- I process how the meeting went on my own.
- I make sure I was left with a good feeling about the group.

**1. Getting to know each other (1½ hours)**

The first meeting is focused on rapport building and becoming aware of personal strengths that are of vital importance for the success of group activity, and form the basis of strength-based coaching. Grouping is supported by letting the group participants come up with the most suitable food-related name for themselves and for the group. Strength cards (<https://pesapuu.fi/>) are used to help participants identify strengths of their own.

**Implementation and setting a timetable for the activities**

**1. Warm-up (~ 40 min)**

1. Welcoming the participants and asking them about how they are feeling about coming to the group today.
2. A short description of the goal and content of the Food for Mind study.

- Getting to know the group process and presenting the timetable for the sessions
- Presenting the Food for Mind portfolio and the dates for small group sessions
- Presenting the key themes of the six small group sessions

1. Getting to know other group members

- Asking everyone to introduce themselves

Independent assignment: Every participant has to give themselves a “team name” based on a food item or ingredient starting with the same initial as their first name.

- Small group name

Pair discussion: The group is divided into pairs. The group names suggested by the pairs are collected on a flip chart and the group selects the best and most fitting suggestion for the group.

- Ground rules for the group

Independent assignment: Handing out a list of the most common ground rules for the group for all participants. Group members select the 5 rules they deem most important by encircling these or add an entry they feel is missing from the list on the blank space reserved for this. A typed-up version of the list is provided to the group members during the following session.

**2. Processing the theme (~ 15 min)**

Participants’ thoughts about the connection between nutrition and depression and how eating affects your mood.

- What was the “incentive” that brought the participants to the Food for Mind study - a discussion.
- Presenting the SMILES study (Jacka FN, O'Neil A, Opie R, Itsiopoulos C, Cotton S, Mohebbi M, et al. A randomised controlled trial of dietary improvement for adults with major depression (the ‘SMILES’ trial). BMC medicine 2017 Jan 30;15(1):23.), Power point - presentation

**3. WhatsApp group – peer support and communication channel (~ 10 min)**

1. Group’s wishes for the WhatsApp group: What sorts of things the participants could share in the group during the group meetings and after the small-group sessions for a 10-month period?
2. “What would it require from the group members to make sure that everyone feels good and safe in the WhatsApp group?” agreeing on ground rules for the WhatsApp group through a discussion (e.g. voluntary participation in the group, limiting the content to topics discussed in the Food for Mind group, having an accepting attitude towards other people’s texts and messages”).

**4. Take home message (~ 10 min)**

Independent assignment: Group members take turns completing the following three sentences:

- ”When I came here today, I felt that…”
- ”The most memorable thing in this session was…”
- ”What I’m taking with me today is…”

**5. Instructions for completing the home assignment (~ 10 min)**

Home assignment

- Food habits that promote wellbeing – ”A good day vs. a bad day”

WhatsApp assignment

- Take a photo of something that is meaningful to you and share it in the group.

**6. Ending the session**

1. Agreeing on the time of the next session and reminding the participants of its theme.
2. Thanking the group members of participating

**2. Diet quality and meal frequency (1½ hours)**

**1. Warm-up (~ 10 min)**

1. Welcoming the group members to the session
2. Reminding the group of an experience from the previous week’s session that was positive or made the group feel good

**2. Discussing the home assignment (~ 20 min)**

1. Independent assignment: The participants write about their experiences of eating on good and bad days on pieces of paper, making use of the assignment they completed at home
2. Discussing the assignment with the whole group: The instructor collects the pieces of paper and writes down the group members’ ideas on a flip chart. Discussion about differences and possible improvement ideas that the participants can think of to the ideas written on the chart.

- What were your most important observations concerning your good days?
- How could you utilize the observations you made on your worse days?

**3. Processing the theme (~ 30 min)**

The session starts with a short conversational Power point presentation on the topic of the meeting, diet quality and meal frequency.

The group members are asked to share their thoughts about the PP presentation concerning the importance of meal patterns and the overall quality of a person’s diet

- Discussing personal experiences and sharing them with the group
- Discussing the importance of focusing on eating and how this can be ensured with the group

**4. Considering wishes for making a change (~ 15 min)**

1. Pair assignment Considering wishes for making a change using a separate handout with exercises, initially in pairs
2. Discussing the assignment with the whole group: Discussing the changes that the group members hope to achieve and how these could be accomplished in the members’ day-to-day lives: making changes concrete, identifying good things and strengthening these.

**5. Take home message (~ 10 min)**

Independent assignment: Group members take turns completing the following three sentences:

- ”When I came here today, I felt that…”
- ”The most memorable thing in this session was…”
- ”What I’m taking with me today is…”

**6. Instructions for completing the home assignment (~ 10 min)**

Home assignment

- Experiment: Focusing on your meal pattern and investing in it for a week
- Home assignment: Assessing personal eating habits

WhatsApp assignment

- Select the starter, main course and dessert that appeal to you most in the Food for Mind - Meals for feeling good recipe booklet, take a photo of these and share the pictures on the WhatsApp group.

**7. Ending the session**

1. Agreeing on the time of the next session and reminding the participants of the theme.
2. Thanking the group members for participating
3. **Nutrition and depression – evidence-based data (1½ hours)**

**1. Warm-up (~ 5 min)**

1. Welcoming the group members to the session
2. Asking the group to recall something they did that made someone else feel good

**2. Discussing the home experiment (~ 15 min)**

1. Pair assignment: Experiences of the home experiment related to meal patterns – observations

-
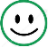
How do you think the smileys are related to eating patterns?

1. Discussing the assignment with the whole group:

**3. Processing the theme (~ 60 min)**

The meeting starts with a short conversational Power point presentation on the topic of the session: Nutrition and Depression.

- The presentation is subsequently discussed together, while recalling the results of the Evaluation of eating patterns home assignment of the Diet quality and meal frequency session.

**4. Take home message (~ 10 min)**

Independent assignment: Group members take turns completing the following three sentences:

- ”When I came here today, I felt that…”
- ”The most memorable thing in this session was…”
- ”What I’m taking with me today is…”

**5. Instructions for completing the home assignment (~ 10 min)**

Home assignment

- Familiarization with the topic of mindful eating before the next session using materials included in the portfolio.
- Experiment: Including an ingredient in your diet that has been suggested to affect depression by some research evidence

WhatsApp assignment

- Take a photo of an ingredient or eating situation that has made you feel good and share the photo in the WhatsApp group.

**6. Ending the session**

1. Agreeing on the time of the next session and reminding the participants of its theme.
2. Thanking the group members for participating

**4. Food for mind in practice (3 hours)**

For the fourth Food for Mind meeting, participants choose from the Food for Mind recipe booklet a starter, main dish, and dessert they want to cook and eat together. The recipe booklet is included in the printed materials to encourage easy cooking at home.

**1. Warm-up (~ 5 min)**

Welcoming the group members to the session

**2. Group assignment: Mindful eating (~ 20 min)**

1. Dividing the group into two groups:

- group 1: mindful eating – what is it?
- group 2: different types of hunger

1. Discussion in a group

- what was the most important observation you made?
- what was new? what surprised you?
- what could I adopt for my personal use also in the future?

**3. Preparing food (~ 75 min)**

Mindfulness exercises – by doing things together and experimenting

- how do you think you could make use of mindfulness at the different stages of cooking (e.g. when chopping vegetables)?
- what does it mean to use all your senses at the different stages of cooking (e.g. when chopping vegetables)?
- a few deep breaths at the start of the exercises and focusing on presence

**4. Eating a meal together (~ 60 min)**

1. A short mindful eating exercise, e.g. in connection with the starter
2. Experiences of adding an ingredient affecting depression according to a new study in your diet – observations made of the home assignment

**5. Take home message (~ 10 min)**

Independent assignment: Group members take turns completing the following three sentences:

- ”When I came here today, I felt that…”
- ”The most memorable thing in this session was…”
- ”What I’m taking with me today is…”

**6. Instructions for completing the home assignment (~ 10 min)**

Home assignment

- A Food for Mind exercise to prepare the participants for the next meeting (a trip to a grocery store); the assignment asks the participants to write down their current food choices.

WhatsApp assignment

- Describe a situation from your daily life where you made use of, of could make use of, the principles of mindfulness, and share it in the WhatsApp group

**7. Ending the session**

- Agreeing on the time of the next session and reminding the participants of its theme.
- Thanking the group members for participating

1. **Food for mind food items in grocery store – navigation (1½ hours)**

The fifth Food for Mind meeting takes place in a grocery store. To prepare for this session, the participants have written down their current food choices.

**1. Gathering at a grocery store and warm-up (~ 5 min)**

Welcoming the group members to the session

**2. Processing the theme (~ 15 min)**

- 1. Introduction: The purpose of the session at the grocery store and its content in brief

1. Group discussion: Experiences and observations of the Food for Mind exercise and discussion on the group member’s personal food choices
2. Providing the participants with an information sheet about the Heart Symbol and presenting it

**3. Visiting the grocery store and discussion (~ 45 min and ~ 20 min)**

- “Which food group did the largest share of items you selected belong to?”
- For fruit and vegetables: “Which of the fruit and vegetables you found were familiar to you? Did you pay attention to different colors? What sort of price variation was there for different fruit and vegetables?”
- “What sort of benefits did you get from this navigation task?”
- “How will you be able to make use of what you learned during this session when visiting a grocery store in the future?”

**4. Take home message (~ 10 min)**

Independent assignment: Group members take turns completing the following three sentences:

- ”When I came here today, I felt that…”
- ”The most memorable thing in this session was…”
- ”What I’m taking with me today is…”

**5. Instructions for completing the home assignment (~ 10 min)**

Home assignment

- Experiment: Focusing on your meal pattern and investing in it for a week

WhatsApp assignment

- Take a photo of a mind-friendly food item you bought after the Food for Mind store navigation session and have used since, and share it with the group.

**6. Ending the session**

1. Agreeing on the time of the next session and reminding the participants of its theme.
2. Thanking the group members of participating

**6. Tools for the future (1½ hours)**

**1. Warm-up (~ 5 min)**

1. Welcoming the group members to the session
2. Observations made from the home experiment – have there been changes to your eating patterns since the previous home experiment concerning eating patterns?

**2. Processing the theme (~ 50 min)**

1. Gallery walk (~ 40 min)

- Dividing participants into three groups (2 – 3 persons/group) – each group starts with a different flip chart

1. Chart 1– SUCCESS (divided into two columns)

- what sorts of experiences of success have you had during the group activities?
- what small concrete changes have you been able to introduce to your day-to-day life?

1. Chart 2 – BENEFIT

- how have you benefited from the changes?

1. Chart 3– SUPPORT (divided into three columns)

- what sort of support have you received from the group?
- what sort of support have you received from others (e.g. friends, family)?
- what means have you found to support your success?

1. Chart 4 – FUTURE

- How will I utilize what I have learned in the Food for Mind group in the future?
- gather in small groups to think about the questions on the flip charts and write your observations and thoughts on the charts (10 min/chart)
- then move on to the next chart, reflect on its topic and write down your thoughts, observations etc.

1. Discussing with the whole group: Discussing and exchanging thoughts

- what sorts of observations did you make of the work/results?
- which issues have helped your success?
- what means do you have at your disposal to make sure that you will also achieve success in the future?

1. Hopefulness and gratitude

- PowerPoint presentation + pair assignment (**~**5 min and **~**5 min)

1. Interactivity during the PP presentation – first asking questions from the group, then addressing them together

- what is hopefulness and how does it show?
- why is hopefulness important?
- how can you increase hopefulness in your day-to-day life?

1. Pair discussion

- what are you grateful about?
- what sorts of thoughts you have when you start thinking about the things you are thankful for?

1. Discussing the topic with the whole group.

**3. Presenting Food for Mind counselling material; future assignments (~ 10 min)**

- Gratitude exercise
- Future letter exercise
- Future resources

**4. Take home message (~ 10 min)**

Independent assignment: Group members take turns completing the following three sentences:

- “When I joined this group, I felt that...”
- “The most memorable thing in this whole group has been...”
- “What I’m taking with me from this group is...”

**5. Handing out diplomas (~ 5 min)**

**6. Feedback questionnaire (~ 10 min)**

**-** Sekhon M, Cartwright M, Francis JJ. Acceptability of healthcare interventions: an overview of reviews and development of a theoretical framework. BMC health services research 2017 Jan 26;17(1):88.
